# Central IKKβ inhibition prevents air pollution mediated peripheral inflammation and exaggeration of type II diabetes

**DOI:** 10.1186/s12989-014-0053-5

**Published:** 2014-10-30

**Authors:** Cuiqing Liu, Laura K Fonken, Aixia Wang, Andrei Maiseyeu, Yuntao Bai, Tse-Yao Wang, Santosh Maurya, Yi-An Ko, Muthu Periasamy, Timothy Dvonch, Masako Morishita, Robert D Brook, Jack Harkema, Zhekang Ying, Bhramar Mukherjee, Qinghua Sun, Randy J Nelson, Sanjay Rajagopalan

**Affiliations:** Department of Physiology, Hangzhou Normal University, Hangzhou, China; Wexner Medical Center, The Ohio State University, Columbus, OH USA; Division of Cardiovascular Medicine, University of Maryland, Baltimore, MD USA; Department of Biostatistics, University of Michigan, Ann Arbor, MI USA; Department of Environmental Health Sciences, University of Michigan, Ann Arbor, MI USA; Center for Integrative Toxicology, Michigan State University, Lansing, MI USA

**Keywords:** Diabetes, Particulate matter, Hypothalamus, Inflammation, IKKβ

## Abstract

**Background:**

Prior experimental and epidemiologic data support a link between exposure to fine ambient particulate matter (<2.5 μm in aerodynamic diameter, PM_2.5_) and development of insulin resistance/Type II diabetes mellitus (Type II DM). We investigated the role of hypothalamic inflammation in PM_2.5_-mediated diabetes development.

**Methods:**

KKay mice, a genetically susceptible model of Type II DM, were assigned to either concentrated PM_2.5_ or filtered air (FA) for 4–8 weeks via a versatile aerosol concentrator and exposure system, or administered intra-cerebroventricular with either IKKβ inhibitor (IMD-0354) or TNFα antibody (infliximab) for 4–5 weeks simultaneously with PM_2.5_ exposure. Glucose tolerance, insulin sensitivity, oxygen consumption and heat production were evaluated. At euthanasia, blood, spleen, visceral adipose tissue and hypothalamus were collected to measure inflammatory cells using flow cytometry. Standard immunohistochemical methods and quantitative PCR were used to assess targets of interest.

**Results:**

PM_2.5_ exposure led to hyperglycemia and insulin resistance, which was accompanied by increased hypothalamic IL-6, TNFα, and IKKβ mRNA expression and microglial/astrocyte reactivity. Targeting the NFκB pathway with intra-cerebroventricular administration of an IKKβ inhibitor [IMD-0354, n = 8 for each group)], but not TNFα blockade with infliximab [(n = 6 for each group], improved glucose tolerance, insulin sensitivity, rectified energy homeostasis (O_2_ consumption, CO_2_ production, respiratory exchange ratio and heat generation) and reduced peripheral inflammation in response to PM_2.5_.

**Conclusions:**

Central inhibition of IKKβ prevents PM_2.5_ mediated peripheral inflammation and exaggeration of type II diabetes. These results provide novel insights into how air pollution may mediate susceptibility to insulin resistance and Type II DM.

## Background

According to the recently published global burden of disease statement, among risk factors, indoor and outdoor air-pollution represents the third and tenth leading causes of global morbidity and mortality respectively [1]. The risks posed by inhaled pollutants are primarily mediated by particulate matter content, especially particles <2.5 μm in aerodynamic diameter (PM_2.5_) [2,3]. Both epidemiologic studies and experimental evidence support adverse cardiometabolic consequences of air-pollution exposure, including worsening of whole body insulin sensitivity, promotion of hepatic endoplasmic reticulum stress, brown adipose dysfunction, and peripheral inflammation [4-6].

Insulin resistance (IR) is widely believed to evolve as a consequence of inflammatory signaling in the peripheral metabolic tissues such as the liver, adipose tissue, skeletal muscle, and/or the vasculature [7-9]. The central nervous system (CNS), particularly the hypothalamus, is a key regulator of metabolic control. In diet-induced obesity, early onset hypothalamic inflammation has been implicated in central resistance to fuel-sensing hormones, loss of homeostatic control of food intake, and changes in energy expenditure. These changes in the hypothalamus have been noted in rodent models, as well as in humans [10-12]. In time course studies, peripheral inflammation takes weeks to develop following initiation of a high fat diet, whereas hypothalamic inflammation occurs rapidly within 24 hrs and prior to substantial weight gain and peripheral inflammation [13,14]. Targeting inflammation in the hypothalamus by the inactivation of IκB kinase or TNFα blockade protects against defective thermogenesis, obesity and IR [15,16], suggesting that hypothalamic dysfunction may precede and mechanistically contribute to obesity-associated IR and Type II diabetes (Type II DM). We hypothesized that inhalation of concentrated PM_2.5_ would result in hypothalamic inflammation and exert effects on peripheral inflammation and IR and tested this hypothesis in a genetically susceptible mouse model of Type II DM.

## Results

### Exposure characteristics

**Figure 1:**
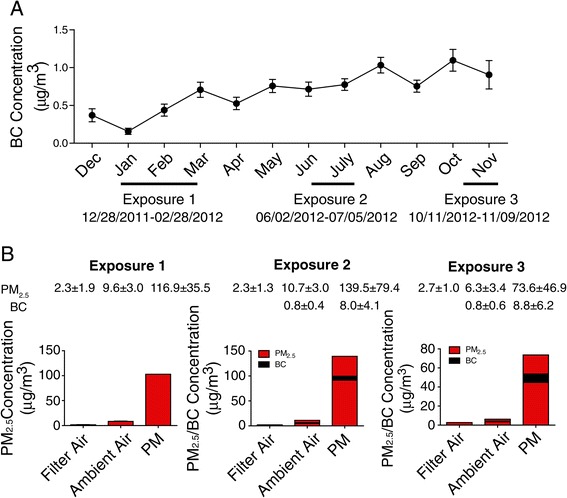
**PM**_**2.5**_ **and BC concentration at the study site. A**, BC concentration in the ambient air from Dec. 2011-Nov. 2012. Exposure 1-Exposure 3 was labeled under the X-axis with exact date information. **B**, PM_2.5_ and BC concentration in the filter air, ambient air and concentrated ambient air for different exposure periods. The mean values of PM_2.5_/BC concentration were shown above the according bar graphs in different exposure period. BC denotes black carbon.

Three batches of KKay mice were exposed by inhalation to either filtered air (FA) or concentrated ambient PM_2.5_ during three distinct exposure periods which were named as Exposure 1, Exposure 2, and Exposure 3 for convenience of description (Figure 1A). During Exposure 1, mice were exposed for 6 h/d, 5 d/wk, 5 weeks or 8 weeks. For Exposure 2, mice were treated (ICV) with infliximab or artificial CSF through an implanted ICV catheter (see ICV drug infusion protocol) and exposed for 6 h/d, 5 d/wk, for 5 weeks. For Exposure 3, mice were treated with IMD-0354 (ICV) or vehicle (DMSO) and exposed for 6 h/d, 5 d/wk, for 4 weeks.

During the three exposure periods, mean concentration of PM_2.5_ was 9.6, 10.7 and 6.3 μg/m^3^ in the ambient air, 2.3, 2.3 and 2.7 μg/m^3^ in the filtered air chamber, 116.9, 139.5 and 73.6 μg/m^3^ in the concentrated PM_2.5_ exposure chamber respectively. This represented 12.2, 13.1 and 11.8-fold concentration over ambient levels respectively (Figure 1B). For the purposes of convenience, concentrated ambient PM_2.5_ exposure is referred to PM_2.5_ in this manuscript unless specified otherwise.

Figure 1A provides the mean concentration of black carbon in the ambient air at the site of exposure facility, for the period Dec. 2011-Nov. 2012. The mean concentration of black carbon in the PM_2.5_ was 8.0 and 8.8 μg/m^3^ for Exposure 2 and Exposure 3 respectively, about 10-fold higher than that of ambient air with 0.8 μg/m^3^ for both exposure periods (Figure 1B). Mean organ carbon and elemental carbon concentrations in the PM_2.5_ for Exposure 3 were 11.5 ± 5.3 μg/m^3^ and 2.0 ± 0.7 μg/m^3^ and together represented 18.9% of concentrated ambient PM_2.5_ mass.

### Exaggeration of IR in KKay mice by PM_2.5_

**Figure 2:**
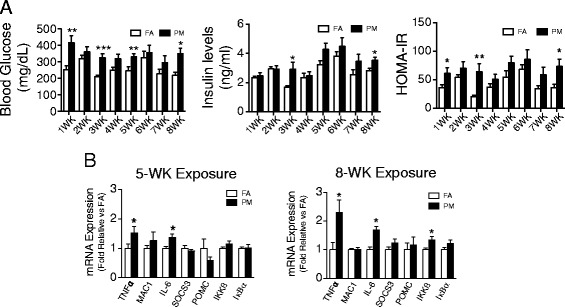
**Effect of PM**_**2.5**_ **exposure on glucose homeostasis and hypothalamic inflammation in KKay mice. A**, Blood glucose, insulin levels and the HOMA-IR after 6-hr fasting during 8 wks of PM_2.5_ exposure. **B**, mRNA levels of inflammation mediators in hypothalamus after 5 wks or 8 wks PM_2.5_ exposure. *P < 0.05, **P < 0.01, ***P < 0.001 compared to respective FA group. n = 6-8 per group.

Since KKay mice are well known to develop abnormalities in glucose metabolism and progressive IR over 5–10 weeks [17], we exposed 5-week old KKay mice to PM_2.5_ for 5 or 8-week periods (Exposure 1) and evaluated them weekly for glucose and insulin measures. No alteration in body weight or food intake was observed during the exposure to PM_2.5_ (data not shown). An increase in fasting blood glucose was seen within 1-week of exposure to PM_2.5_, while insulin levels trended upwards, with the highest values after 6-week of exposure and significant differences between the two groups at the 3- and 8-week time-points (Figure 2A). Corresponding homeostasis model assessment of the IR index (HOMA-IR) levels were significantly different at the 1-, 3- and 8-week time-points (Figure 2A).

### PM_2.5_ exposure induces inflammation in hypothalamus of KKay mice

**Figure 3:**
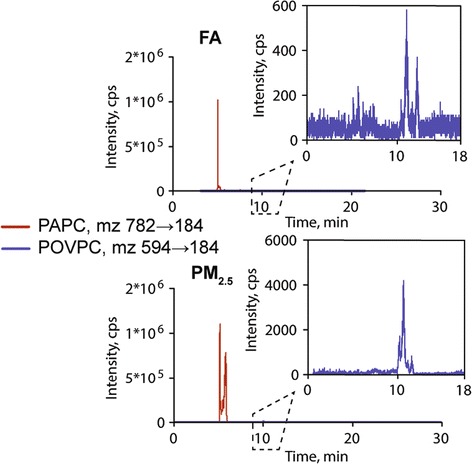
**Representative LC** **-MS chromatograms showing effect of PM**_**2.5**_ **exposure on oxidized PAPC in brain of mice.** Lipid extracts from brain of mice exposed to FA or PM_2.5_ were analyzed by HPLC with positive electrospray ionization mass spectrometry operating in multiple reaction monitoring mode.

Air pollution has been previously shown to cause CNS inflammation, oxidative stress and pathological alterations such as reactive gliosis [18,19]. Our group has reported that long term exposure to PM_2.5_ over 10 months results in hippocampal pro-inflammatory cytokine expression [20]. Although hypothalamic inflammation is well-documented in models of diet-induced obesity [10,15,21], its role as a potential mediator of altered energy and glucose homeostasis in response to air-pollution has not been explored. To address this question, we examined mRNA encoding inflammatory mediators, including cytokines (IL-6, TNFα), Suppressor of cytokine signaling 3 (SOCS-3), components of the NFκB pathway (IKKβ and IκB), and microglia/macrophage (MAC1). In the hypothalamus of mice exposed to PM_2.5_, TNFα and IL-6 expression was elevated after 5-week of exposure to PM_2.5_ as compared to FA (Figure 2B). Longer duration (8-week) exposure to PM_2.5_ elevated TNFα and IL-6 expression, as well as significantly increased IKKβ expression. There was no difference in the mRNA levels of other genes (Figure 2B). These results suggest that even short-term exposure of a few weeks is sufficient to induce increases in cytokine expression in the medial basal hypothalamus. To further understand the mechanisms responsible for inflammation, we assessed the levels of oxidized 1-palmitoyl-2-arachidonoyl-sn-glycero-3-phosphocholine (oxPAPC), a prototypic biologically active oxidized phospholipid in the brains of mice exposed to PM_2.5_ and compared these to FA exposed mice. Ox-PAPC has been previously shown by us and others to activate NFκB pathways via toll-like receptor pathways [22,23]. The level of oxPAPC was elevated in PM_2.5_ exposed mice compared to controls (286054 vs. 180579 arbitrary units). When expressed as a % of PAPC, the PM_2.5_ exposed animals demonstrated a 2-fold increase in ox-PAPC/PAPC ratio compared to FA exposed mice (Figure 3).

### ICV infliximab effects on PM_2.5_-mediated effects on metabolism parameters

**Figure 4:**
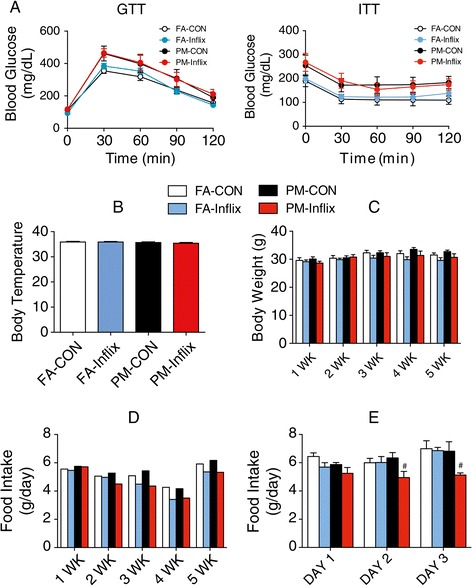
**Metabolism response to intracerebroventricular infusion of Infliximab in KKay mice exposed to PM**_**2.5**_ **. A**, GTT and ITT of PM_2.5_ exposed KKay mice after 5-week treatment with TNFα antibody. **B**, Average rectal temperatures for mice by the end of 5-week PM_2.5_ exposure. **C**, Body weight of mice during 5-week PM_2.5_ exposure. **D**, Average daily food consumption during **(D)** or after **(E)** 5-week PM_2.5_ exposure. *P* < 0.05 when compared with PM-CON group.

Based on the increased hypothalamic TNFα expression in PM_2.5_ mice and observations that this may contribute to changes in peripheral inflammation including brown adipose tissue dysfunction [24], we hypothesized that TNFα antagonism may restore peripheral glucose intolerance and altered thermogenesis following PM_2.5_ exposure. Mice at age of 5-week were continuously administered infliximab (0.2 μg/day) or artificial cerebrospinal fluid (aCSF) through a minipump connected to a cannula directed at the lateral ventricle. Minipumps were implanted 1 day prior to initiation of either PM_2.5_ or FA exposure (Exposure 2, Figure 1B). As shown in Figure 4A, intracerebroventricular (ICV) infliximab did not influence peripheral glycemia or insulin tolerance in response to PM_2.5_ exposure; neither did body temperature, body weight differ between groups (Figure 4B-4C). However food intake was lowered in the PM-Inflix group compared to PM-CON both during the exposure period (Figure 4D) or after the exposure period (Figure 4E).

When analyzing the energy parameters, we used two different analytical strategies as detailed in the Methods. In the first analysis, we used the average measure for each index over time for each mouse and used linear regression. Using this approach no significant difference in O_2_ consumption, CO_2_ production, respiratory exchanging ratio (RER) or heat production was observed in response to PM_2.5_ exposure (Table 1). However, infliximab treatment inhibited heat production in PM_2.5_ inhaled mice regardless of phase of day. Using repeated measures analysis (adjusting for time), much more significant differences were found. Although only O_2_ consumption and CO_2_ production were inhibited in response to PM_2.5_ inhalation when the data for the entire day were used, PM_2.5_ inhalation additionally inhibited heat generation during the dark phase (Table 1). These results indicate a consistency of effect of PM_2.5_ and additionally that metabolism itself displays circadian variation with a higher activity during the dark phase and lower activity during light phase for rodents. Infliximab treatment significantly impaired energy homeostasis, as evidenced by further decreases in O_2_ consumption, CO_2_ production, RER, and heat generation in infliximab-treated mice compared to controls (Table 1).

**Table 1:** **Energy metabolism response to intracerebroventricular infusion of infliximab in KKay mice exposed to PM**_**2.5**_

**Phase**	**Measured index**	**Method**	**FA-CON vs PM-CON**			**PM-CON vs PM-Inflix**		
			**Estimate**	**SE**	**p-value**	**Estimate**	**SE**	**p-value**
Light + Dark	O_2_	Avg	−193.125	170.601	0.270	−182.738	170.601	0.296
		RM	−171.714	66.509	0.010	−184.556	66.509	0.006
	CO_2_	Avg	−186.684	197.569	0.355	−248.623	197.569	0.222
		RM	−159.923	79.478	0.044	−248.577	79.478	0.002
	RER	Avg	−0.001	0.015	0.969	−0.025	0.015	0.113
		RM	0.001	0.008	0.899	−0.025	0.008	0.003
	Heat	Avg	−0.020	0.033	0.548	−0.076	0.033	0.030
		RM	−0.016	0.012	0.204	−0.078	0.012	0.000
Dark	O_2_	Avg	−272.695	195.576	0.178	−186.257	195.576	0.352
		RM	−278.074	85.804	0.001	−186.729	85.804	0.030
	CO_2_	Avg	−273.400	234.828	0.257	−289.425	234.828	0.231
		RM	−280.245	106.432	0.009	−287.488	106.432	0.007
	RER	Avg	−0.002	0.019	0.901	−0.037	0.019	0.062
		RM	−0.003	0.010	0.753	−0.036	0.010	0.000
	Heat	Avg	−0.035	0.035	0.338	−0.079	0.035	0.036
		RM	−0.036	0.016	0.022	−0.079	0.016	0.000

O_2_ consumption, CO_2_ production, RER and heat production of mice measured by indirect calorimetry over a 24 hrs period (from 10:00 am to 10:00 am the next day) after 5-week PM_2.5_ exposure. n = 6.

*Avg* = analysis performed based on mice averages over cycles. Covariates in the model include PM exposure, treatment group, and the interaction between PM and treatment group.

*RM* = analysis with repeated measurements. The correlation structure within mice was assumed to be autoregressive-1. Covariates in the model include PM, treatment group, PM x treatment interaction, and time.

Mean model using averages over cycles (Avg): E (Y) = b0 + b1PM + b2 Trt + b3 PM*Trt.

Mean model using repeated measures data (RM): E (Y + b1P) = b0 M + b2 Trt + b3 PM*Trt + b4cycle.

Covariance structure: autoregressive-1.

### Central IKKβ inhibition prevents PM_2.5_-induced disruption of metabolism

**Figure 5:**
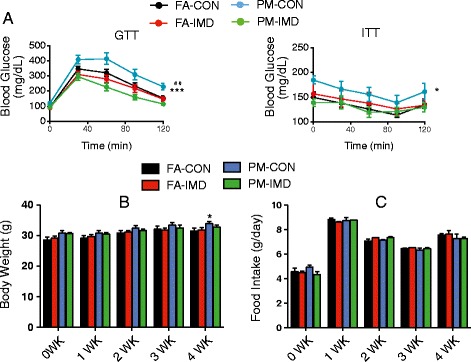
**Glucose homeostasis response to ICV infusion of IMD-** **0354 in KKay mice exposed to PM**_**2.5**_ **. A**, GTT and ITT of PM_2.5_ exposed KKay mice after 4-week treatment with IMD-0354. **B**, Body weight during the 4-week of PM_2.5_ exposure and IKKβ inhibitor treatment. **C**, Food intake during the 4-week of PM_2.5_ exposure and IKKβ inhibitor treatment. *P < 0.05, ***P < 0.001 compared to FA-CON group; ##*P* < 0.01 when compared PM-IMD group with PM-CON group. n = 6-8 per group.

To determine whether upregulated hypothalamic IKK-NF-κB pathway contributes to PM_2.5_-exaggarated IR, we continuously administered IMD-0354, a pharmacological inhibitor of IKKβ (IKK2) through a minipump connected to a cannula directed at the lateral ventricle in the same way as Infliximab treatment. Minipumps were implanted 1 day prior to initiation of either PM_2.5_ or FA exposure for 4 weeks (Exposure 3, Figure 1). Consistent with the results from “exposure 1”, PM_2.5_ induced abnormal glucose tolerance and attenuation of whole-body insulin sensitivity, which were normalized with IMD-0354 treatment (Figure 5A). This effect was PM_2.5_ dependent, as IMD-0354 did not further improve these parameters in the FA group (Figure 5A). No other difference was shown either in body weight or in food intake during the exposure period between groups except for an increase in body weight after 4 weeks PM_2.5_ exposure (Figure 5B-C). Taken together, these results suggest that central IKKβ inhibition prevents PM_2.5_-induced abnormalities in glucose/insulin homeostasis.

**Figure 6:**
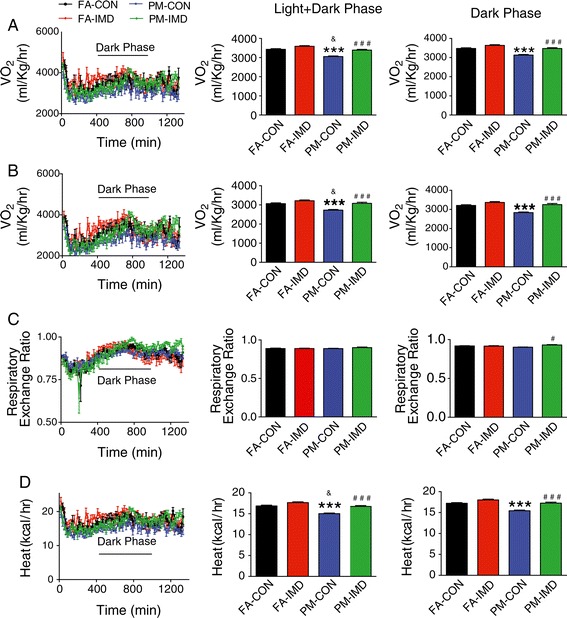
**Energy metabolism response to ICV infusion of IMD-** **0354 in KKay mice exposed to PM**_**2.5**_ **.** O_2_ consumption **(A)**, CO_2_ production **(B)**, respiratory exchanging ratio **(C)** and heat production **(D)** of mice measured by indirect calorimetry over a 24 hrs period (from 10:00 am to 10:00 am the next day) after 4 wks of PM_2.5_ exposure and IKKβ inhibitor treatment. When RM (repeated measures adjusting for time) analysis was applied: ***P < 0.001 compared to FA-CON group; #*P* < 0.05, ###*P* < 0.001 when compared PM-IMD group with PM-CON group. When Avg (the average over time followed by linear regression) was applied, &*P* < 0.05 compared to FA-CON group. n = 6 per group.

To examine whether central IKKβ Inhibition could improve PM_2.5_-induced impairment of energy metabolism, we measured O_2_ consumption, CO_2_ production, RER and heat production and analyzed the data with two analytical strategies. When repeated measures analysis was applied, PM_2.5_ inhalation inhibited energy metabolism, reflected by decreased O_2_ consumption, CO_2_ production and heat generation, both in the whole day phase and in the dark Phase (Figure 6A,B,D). As expected, ICV IMD-0354 normalized the energy metabolism, evidenced by restoration of these parameters (O_2_ consumption, CO_2_ production heat generation both in the whole day phase and in the dark phase) and further increase in RER in the dark phase, compared with PM_2.5_-exposed mice administered vehicle (Figure 6 and Table 2). When the analysis of average over time followed by linear regression was applied, O_2_ consumption, CO_2_ production and heat generation were all inhibited in response to PM_2.5_ inhalation only during the day but not the dark period (Figure 6A,B,D). No significant difference was observed between IMD-0354 treated groups and their respective controls (Figure 6, Table 2).

**Table 2:** **Energy metabolism response to intracerebroventricular infusion of IMD 0354 in KKay mice exposed to PM**_**2.5**_

**Phase**	**Measured index**	**Method**	**FA** **-CON vs PM** **-CON**			**PM-** **CON vs PM-** **IMD**		
			**Estimate**	**SE**	**p-** **value**	**Estimate**	**SE**	**p** **-value**
Light + Dark	O_2_	Avg	−370.927	149.129	0.032	331.662	167.254	0.061
		RM	−371.022	59.968	0.000	335.500	64.236	0.000
	CO_2_	Avg	−333.440	127.020	0.025	347.582	181.272	0.070
		RM	−332.649	62.399	0.000	351.053	76.737	0.000
	RER	Avg	0.000	0.009	0.985	0.011	0.016	0.473
		RM	0.000	0.006	0.989	0.011	0.009	0.205
	Heat	Avg	−1.826	0.722	0.030	1.694	0.855	0.061
		RM	−1.825	0.303	0.000	1.712	0.336	0.000
Dark	O_2_	Avg	−346.732	185.106	0.076	340.550	185.106	0.081
		RM	−340.496	82.903	0.000	336.695	82.903	0.000
	CO_2_	Avg	−372.246	209.234	0.090	414.139	209.234	0.062
		RM	−359.378	97.723	0.000	406.893	97.723	0.000
	RER	Avg	−0.016	0.017	0.360	0.028	0.017	0.117
		RM	−0.012	0.010	0.248	0.026	0.010	0.012
	Heat	Avg	−1.781	0.958	0.078	1.809	0.958	0.073
		RM	−1.743	0.433	0.000	1.786	0.433	0.000

O_2_ consumption, CO_2_ production, RER and heat production of mice measured by indirect calorimetry over a 24 hrs period (from 10:00 am to 10:00 am the next day) after 5-week PM_2.5_ exposure. n = 6.

Avg = analysis performed based on mice averages over cycles. Covariates in the model include PM exposure, treatment group, and the interaction between PM and treatment group.

RM = analysis with repeated measurements. The correlation structure within mice was assumed to be autoregressive-1. Covariates in the model include PM, treatment group, PM x treatment interaction, and time.

Mean model using averages over cycles (Avg): E (Y) = b0 + b1PM + b2 Trt + b3 PM*Trt.

Mean model using repeated measures data (RM): E (Y) = b0 + b1PM + b2 Trt + b3 PM*Trt + b4cycle.

Covariance structure: autoregressive-1.

### Central IKKβ inhibition ameliorates PM_2.5_-induced peripheral inflammation

**Figure 7:**
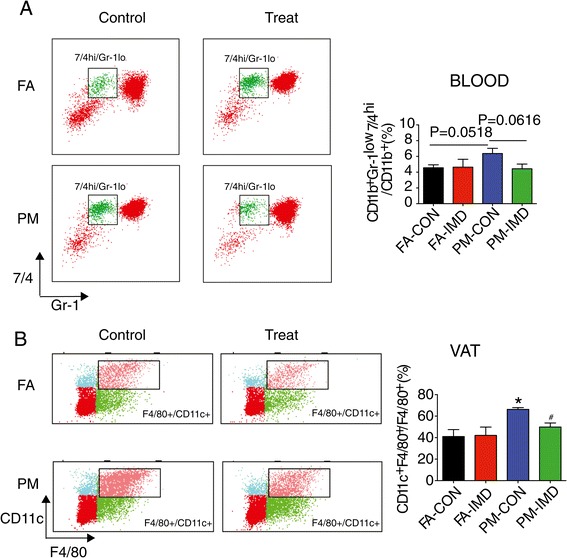
**Anti-** **inflammation in blood and VAT in response to ICV IMD-** **0354 in KKay mice exposed to PM**_**2.5**_ **. A**, Representative flow cytometric dot plots and analysis showing circulating CD11b^+^Gr-1^low^7/4^hi^ cells from mice blood at the end of 4-week PM_2.5_ exposure and IMD-0354 treatment. **B**, Representative flow cytometric dot plots and analysis showing CD11c^+^F4/80^+^ cells from VAT at the end of 4 week PM_2.5_ exposure and IMD-0354 treatment. *P < 0.05 compared to FA-CON group, #*P* < 0.05 when compared PM-IMD group with PM-CON group. n = 6-8 per group.

We next investigated the effect of central IKKβ inhibition on peripheral inflammation induced by PM_2.5_ exposure. In the present study, we defined monocytes as side scatter-high, forward scatter-low cells expressing the myeloid antigen 7/4 (high populations) and high levels of CD11b but low for the neutrophil marker Gr-1 (Ly6G), which corresponds to Ly6C^hi^ monocytes and represents the inflammatory subtype [23,25,26]. We noted a clear trend (P = 0.0518) of increase in circulating CD11b^+^Gr-1^low^7/4^hi^ cells, the inflammatory subtype in response to PM_2.5_ exposure. The levels of CD11b^+^Gr-1^low^7/4^hi^ in circulation were reduced in mice treated with IMD-0354 ICV (Figure 7A).

F4/80^+^CD11c^+^ is a macrophages marker and these cells have been demonstrated to play a pathophysiological role in high-fat diet-induced obesity [27-31]. F4/80^+^/CD11c^+^ cells in visceral adipose tissue (VAT) were markedly higher in response to PM_2.5_ exposure, with the increase being attenuated by IMD-0354 treatment (Figure 7B). Together with data demonstrating that PM_2.5_-mediated monocytes infiltration into VAT is CCR2 dependent [32], these results suggest mechanisms similar to those involved in diet mediated aggravation of the VAT infiltration by monocytes via CCR2 dependent pathways [29]. These results indicate that peripheral inflammation in blood and adipose tissue in response to PM_2.5_ is dependent on CNS inflammation to a considerable degree.

### Central IKKβ inhibition suppresses PM_2.5_-induced hypothalamic inflammation

**Figure 8:**
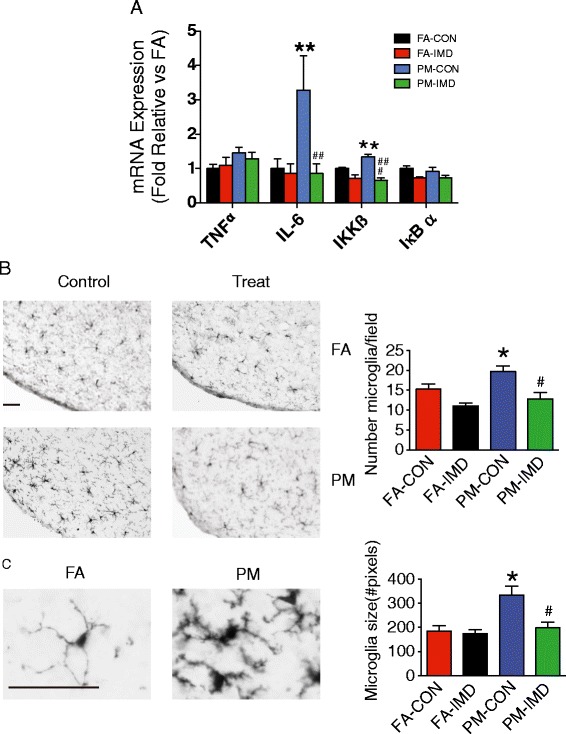
**Hypothalamic inflammatory genes and microglia in response to ICV infusion IMD** **-0354 in KKay mice exposed to PM**_**2.5**_ **. A**, mRNA for proinflammatory cytokines (*TNFα and IL*-*6*) and NF-κB pathway (*Ikkβ* and *IκBα*) gene expression in the hypothalamus of mice exposed to PM_2.5_ and treated with an IKKβ inhibitor for 4 weeks. **B**, Immunohistochemical detection of Iba1 protein, a microglial marker. Representative photomicrographs captured at 20X from KKay mice treated with vehicle or received IKKβ inhibitor (bar = 50 μm). ARC microglia cell number was quantified and normalized to each visual field. **C**, Representative image (40X) of microglial fine structure in the hypothalamus of vehicle-treated mice exposed to FA and PM_2.5_ (bar = 50 μm). Mean microglia size was quantified by average number of pixels in 5 representative cells. Data are expressed as mean ± SEM. *P < 0.05 compared to FA-CON group, ^#^ *P* < 0.05 when compared with PM-CON group. n = 7-8 per group.

**Figure 9:**
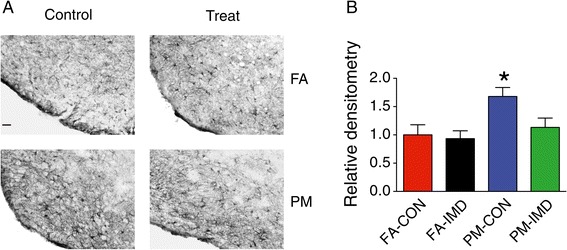
**Hypothalamic astrocytes in response to intracerebroventricular infusion of IMD-** **0354 in KKay mice exposed to PM**_**2.5**_ **. A**, Representative images (10X) of astrocytes identified by immunohistochemical detection of GFAP protein in the hypothalamus of PM_2.5_-exposed mice treated with IKKβ inhibitor. The bar = 50 μm. **B**, Quantification of GFAP staining intensity in the ARC. Data are expressed as mean ± SEM, *P < 0.05 when compared with FA-CON group.

As depicted in Figure 8A, IMD-0354 reduced IL-6 and IKKβ expression, both of which were up-regulated by PM_2.5_ exposure, but had no effect on TNFα and IκB expression. Reactive gliosis, identified by the recruitment, activation, and proliferation of glial cells, such as astrocytes, NG2 cells, and microglia, in the arcuate nucleus of the hypothalamus, has been associated with changes in metabolic homeostasis [13]. Thus, we investigated the effects of PM_2.5_ exposure and IKKβ inhibition on microglia and astrocyte reactivity in the arcuate nucleus of the hypothalamus. Using anti-Iba1 (ionized calcium binding adaptor molecule 1), a microglia-specific cytoplasmic marker [33], we found a 20% increase in microglial number in the arcuate nucleus of mice exposed to PM_2.5_ compared to FA (Figure 8B). Additionally, microglia from PM_2.5_ mice were larger with a more activated morphology (Figure 8C). Central IMD-0354 infusion restored both the number and size of microglia in response to PM_2.5_ (Figure 8B-C). The effect of PM_2.5_ on astrocytes was assessed with GFAP immunostaining. Astrocytes are abundant throughout the CNS and were apparent in the arcuate nucleus of all groups. The intensity of GFAP staining which was elevated among PM_2.5_ mice was prevented by treatment with the IKKβ inhibitor (Figure 9). Chronic high fat feeding results in prolonged inflammatory responses in the CNS, which can cause loss of sensing in proopiomelanocortin (POMC) neurons [34]. POMC neurons are critical components of the network regulating energy balance in mammals and loss of POMC neurons is associated with development of metabolic syndrome [35]. Therefore we examined evidence of POMC neuronal loss in the hypothalamus; however, neither PM_2.5_ exposure nor drug treatment affected POMC number (data not shown, *P* > 0.05).

## Discussion

In this paper we demonstrate PM_2.5_ exposure induces hypothalamic inflammation in a genetically susceptible model of Type II DM. The increased cytokine expression in the hypothalamus was accompanied by evidence of exacerbation of peripheral glycemia and IR. Increased levels of oxidized phospholipids in the brain may represent one possible mechanism that may account for activation of downstream inflammatory pathways. Central IKKβ inhibition but not TNFα blockade prevented the effects of PM_2.5_ on glucose tolerance and insulin sensitivity, restored abnormal O_2_ consumption, CO_2_ production, heat generation and inhibited the PM_2.5_-enhanced peripheral inflammation. Importantly, central IKKβ inhibition effectively reduced IL-6 and reactive gliosis in hypothalamus. In addition, the consistency of the effect regardless of location of exposure (urban in exposure 1 vs. near roadway in exposures 2 and 3) suggests that there may be no significant differences at least within the confines of a large urban area, regardless of proximity to a highway, although our study was not designed to test these differences.

A number of studies have shown that exposure to PM_2.5_ is associated with, or lead directly to IR, adiposity, plaque destabilization, and adverse cardiovascular events, in which pro-inflammatory and oxidative stress pathways play critical roles [5,36-40]. In rodent models of diet-induced obesity, increased inflammatory signaling in the mediobasal hypothalamus occur early and prior to peripheral inflammation and altered energy homeostasis [12,13,41]. These findings suggest that inflammatory changes in the CNS may mechanistically contribute to the development of obesity and IR [15].

The CNS plays a critical role in energy balance with multiple environmental and internal signals serving as cues to trigger the requisite behavioral and physiological response to maintain energy homeostasis. Considerable progress has been made in elucidating the molecular and cellular pathways, primarily within the hypothalamus and hindbrain. In previous studies, we have demonstrated evidence of CNS inflammation in the hippocampus with long term PM_2.5_ exposure (10 months) [20]. However, the results in the present study suggest that exposure even over a few weeks is sufficient to induce increase in TNFα and IL-6 and reactive gliosis in the medial basal hypothalamus. In this regard, the hypothalamus may be particularly vulnerable to the effects of diet and environmental signals such as PM_2.5_ because the blood brain barrier is relatively permeable in this part of the brain.

Our findings may have important implications for potential pathways by which PM_2.5_ mediate alteration in peripheral metabolic dysfunction. Examining inflammation in hypothalamus, even earlier (within the first several days of PM_2.5_ inhalation), would help to clarify the initiating role of central inflammation in the genesis of IR. PM_2.5_ has been shown to permeate the CNS via translocation along the olfactory nerve into the olfactory bulb and exert direct effects on CNS inflammation [19,42]. Alternatively, PM_2.5_ exposure may directly affect vagal afferents that may play an important role in modulation of pathways that affect cardiovascular and/or peripheral inflammatory responses [43]. As alterations in hypothalamic signaling induced by overnutrition (particularly hypothalamic leptin resistance) has been demonstrated to alter sympathetic outflow, this mechanism could also help couple sympathetic activation with systemic IR [44-46]. In addition, neurotransmitters released through peripheral and autonomic nerves may play an important role in activation of these cells, thus linking the alterations in neural function with peripheral inflammation [47]. However, additional studies are required to characterize the precise pathways.

In light of the importance of hypothalamic TNFα in response to high fat diet [10] and our own results of the increased TNFα, we hypothesized that this cytokine may play a role in PM_2.5_-mediated IR and that its inhibition may prevent progression of IR in the KKay model. However, TNFα blockade at the dosage used in the study did not antagonize the adverse effects of PM_2.5_ on glucose metabolism and energy homeostasis. Our results are however contrary to reports showing that central TNFα administration reduced the expression of thermogenic proteins in brown adipose tissue and skeletal muscle, effects that were blunted in TNFα receptor knockout mice [16,48]. Depending on its local concentrations, TNFα can exert dual functions in the hypothalamus, being catabolic at high and anabolic at low concentrations. Consistent with these divergent effects on energy metabolism, TNFα levels in obese animals are higher than that in control rats but significantly lower than that in tumor-bearing rats. These effects were accompanied by inhibition of feeding/anorexia in tumor-bearing, and increase in feeding (orexigenic effect) in obese animals respectively [16]. It is attractive to propose that the effect of infliximab, is a reflection of the paradoxical, concentration dependent effect of TNFα on metabolism.

In contrast, antagonism of IKKβ, the enzyme regulating cytokine production, completely corrected PM_2.5_-induced dysfunction of glucose homeostasis. These effects are consistent with a role for NFκB activation via toll-like receptor mechanisms in response to oxPAPC generated by PM_2.5_ as previously demonstrated by us [23]. This mechanism while applicable to PM_2.5_, may potentially apply to other situations including high-fat feeding. Consistent with this, Zhang et al. have previously demonstrated increased expression and activation of hypothalamic IKKβ/NF-κB in obesity, both in leptin-deficient ob/ob mice fed a normal chow and in high fat diet-induced obese animals [15]. These results suggest that hypothalamic IKKβ but not TNFα is important in PM_2.5_-mediated peripheral effects and suggest a role for TLR pathways (at least not TNFα) pathways in PM_2.5_ mediated peripheral effects.

Although our results suggest an important role for CNS inflammation via IKKβ in PM_2.5_-mediated diabetes development, several questions remain. Firstly, it is not known which cell type(s) are involved in the initiation of PM_2.5_-induced inflammatory responses in hypothalamus. Although evidence suggests that IKKβ/NF-κB in specific neuronal populations is critical to high fat diet-induced inflammation and IR, the precise cell type and the role of microglial cells remain unclear [15,49]. Secondly, the specific components of PM_2.5_ that mediate these effects remain to be defined. We have provided detailed analysis on components such as trace elements in prior studies [32,50] as well as organic carbon fraction in this study (Figure 1). We however submit that source apportionment type analysis is clearly beyond the scope of this paper. Thirdly, we can not exclude direct off target systemic effects with the pharmacological inhibitor of IKKβ by ICV in the study. To further confirm the hypothesis, targeted deletion of IKKβ, either with direct intra-nuclear injection into the arcuate nucleus of adenovirus expressing dominant negative IKKβ or cre-recombinase in conditionally expressed models will be required. However, considering that the dose used in the present study (600 ng/day ICV) was about 1,000-fold lower than that used when peripherally administered (10–20 mg/kg), it is unlikely that the effects seen in this study are occurring secondary to systemic spill-over [51]. So, even there is some “spillover”, it is very unlikely to contribute to systemic effects. Fourthly, the precise upstream pathways leading to the activation of IKKβ and the precise molecular instigators of IKK-β activation remain to be determined.

In summary, our results suggest an important role for PM_2.5_-induced hypothalamic inflammation in modulating susceptibility to Type II DM. Given the importance of air pollution as a mediator of global morbidity and mortality, its continuous and omnipresent nature, even small adverse health associations for individuals may have profound public health implications on a global scale [1,2].

## Methods

### Animals and animal care

KKay mice of 5-week-old or 7-week-old were purchased from Jackson Laboratories (Bar Harbor, Me). All mice were maintained at 21°C on a 12-h light/12-h dark cycle with free access to water and food. The protocols and the use of animals were approved by and in accordance with the Ohio State University Animal Care and Use Committee. The animals were treated humanely and with regard for alleviation of suffering.

### Ambient whole-body inhalational protocol and groups

There were three distinct exposure periods, Exposure 1, Exposure 2, and Exposure 3. During the exposure periods, KKay mice were exposed by inhalation to either FA or concentrated ambient PM_2.5_ with 2 different exposure systems (“Ohio Air Pollution Exposure System for Interrogation of Systemic Effects”) located either at the Ohio State University Laboratory Animal Center (LAC) Facility in Godown Road Columbus (urban exposure facility, not proximal to a major roadway) or at the Polaris Facility (near roadway facility located within 250 m of a major interstate highway). Exposure 1 was conducted in LAC while Exposure 2 and 3 were conducted in Polaris Facility. For Exposure 1 (mice were exposed for 6 h/d, 5 d/wk, 5 weeks or 8 weeks. For Exposure 2, mice were treated (ICV) with infliximab or artificial CSF through an implanted ICV catheter (see ICV drug infusion protocol) and exposed for 6 h/d, 5 d/wk, for 5 weeks. For Exposure 3, mice were treated (ICV) with IMD-0354 or vehicle of DMSO and exposed for 6 h/d, 5 d/wk, for 4 weeks. For ease of identification, the animal groups were named 5WK-FA, 5WK-PM, 8WK-FA and 8WK-PM during Exposure 1 (n = 7-8/group); FA-CON and PM-CON (ICV with aCSF), FA-Inflix and PM-Inflix (ICV with TNFα antibody, Infliximab, Remicaded), during Exposure 2 (n = 6/group); FA-CON and PM-CON (ICV with vehicle of DMSO), FA-IMD and PM-IMD (ICV with IKKβ inhibitor, IMD-0354, Sigma) during Exposure 3 (n = 8 for each group). Animal exposure and monitoring of the exposure environment and ambient aerosol were performed as previously described [5,36].

### PM_2.5_ concentration in the exposure chamber

To calculate exposure mass concentrations of concentrated ambient PM_2.5_ in the exposure chambers, samples were collected on Teflon filters (PTFE, 37 mm, 2 μm pore; PALL Life Sciences, Ann Arbor, MI) and weighed before and after sampling in a temperature- and humidity-controlled weighing room using a Mettler Toledo Excellence Plus XP microbalance. Weight gains were used to calculate exposure concentrations.

### ICV drug infusion

A stereotaxic apparatus was used to implant a cannula into the right lateral ventricle of mice anesthetized with 2% isoflurane in air. Cannula positions were +0.02 posterior and −0.95 lateral to Bregma, extending 2.75 mm below the skull (Plastics One, Roanoke, VA). The cannula was connected via tubing to an Alzet minipump (Model 1004, Durect, Cupertino, CA) that was implanted subcutaneously in the scapular region and delivered either drugs (Infliximab and IMD 0354) or vehicles, both at a rate of 0.11 μL/h. Minipumps were implanted 1 day prior to initiation of either PM_2.5_ or FA exposure. The infliximab-treated groups received a total of 0.2 μg of the antibody, and the IMD-0354-treated groups received a total of 600 ng of the inhibitor per day. Cannula placement was verified in tissue used for immunohistochemistry.

### Measurements of blood glucose homeostasis and insulin sensitivity

Before and subsequent to the exposure to FA or PM_2.5_, mice were fasted overnight and dextrose (2 mg/g body weight) was injected intra-peritoneally for intra-peritoneal glucose tolerance testing (IPGTT). Blood sample was collected from the vena caudalis and blood glucose measurement was conducted with an Contour Blood Glucose Meter (Bayer, Mishawaka, IN) at baseline, and 30, 60, 90, and 120 minutes after the dextrose injection. During the exposure period, 6 h-fasting blood glucose was monitored every week, with the same glucose meter as GTT. Insulin levels were determined using an Ultra Sensitive Mouse Insulin ELISA Kit (Crystal Chem Inc., Downers Grove, IL). HOMA-IR were calculated based on 1 mg of insulin as equivalent to 24 IU, using the formula HOMA = [fasting insulin concentration (ng/ml) × 24 × fasting glucose concentration (mg/dl)]/405 [36].

By the end of exposure, insulin sensitivity was measured by the insulin tolerance test (ITT). After 4.5 hours fasting, Insulin (0.5 U/kg) was administered by intra-peritoneal injection. Blood glucose measurement was conducted in the same way as IPGTT with the same Contour Blood Glucose Meter at baseline, and 30, 60, 90, and 120 minutes after insulin injection.

### Oxygen consumption and heat production measurement

The mice were isolated in a semi-sealed cage, and the inner air was aspirated at a constant volume/min. Oxygen consumption, CO_2_ production, respiratory exchanging ratio and heat production were measured simultaneously using a computer-controlled, open-circuit Oxymax/CLAMS System (Columbus Instruments, Columbus, OH). Each mouse was measured individually in a resting state at 22°C in the presence of food and water [52]. Measurements were taken for a 24-h period, including a 12-h light cycle and a 12-h dark cycle. Data were normalized to body weight.

### Flow cytometric evaluation of inflammation in blood/tissues

Visceral adipose tissues from the mice were excised, minced, and digested with collagenase type II, and the SVF isolated as described previously.These cells were centrifuged at 500 × g for 5 min. Whole blood was centrifuged at 500 × g, 4°C for 5 min and plasma was collected. The remaining blood cells and the resulting pellets were re-suspended in 1X red blood cell lysis buffer (Biolegend, San Diego, CA), at room temperature for 3 minutes followed by addition of 1 X PBS and centrifugation. Then, blood cells spleen cells and bone marrow derived cells were stained with anti-CD11b, anti-7/4 and anti-Gr-1, SVFs were stained with anti-CD11c and F4/80, both followed by incubation at room temperature for 45 minutes. Cells were subsequently washed with 1 X PBS and re-suspended in 1% neutral buffered formalin and run by flow cytometry (BD FACS LSR II™ flow cytometer, Becton Dickinson, San Jose, CA). Data was analyzed using BD FACS Diva software (Becton Dickinson, San Jose, CA). All antibodies were purchased from Biolegend, Miltenyi Biotec, or BD Bioscience [23,53].

### Immunohistochemistry

Mice were perfused transcardially with ice-cold 0.1 *M* PBS. Brains were removed, divided at the hemisphere with a sterile razor, and the right hemisphere was fixed overnight in 4% paraformaldehyde. The half brains were subsequently cyroprotected in 30% sucrose, frozen in isopentane with dry ice, and stored at −80°C. Eighteen μm brain sections were sliced at −22°C using a cryostat, thaw mounted onto Super Frost Plus slides (Fisher, Hampton, NH), and stored at −20°C. The sections were rinsed in PBS and blocked with 4% BSA in PBS + Triton-X (TX) for 1 h with constant agitation. Alternate slides were incubated overnight with rabbit anti-Iba-1 (1:1000, Wako Chemicals, Richmond, VA), rabbit anti-GFAP (1:1000, abcam, Cambridge, MA) or rabbit anti-POMC (1:4000; Pheonix Pharmaceuticals, Burligame, CA). After PBS rinses the slides were subsequently incubated for 1 h at room temperature with biotinylated goat-anti-rabbit 1:1000 in PBS + TX (Vector Laboratories, Burligame, CA). Sections were then quenched for 20 min in methanol containing 0.3% hydrogen peroxide. After washing with PBS, sections were incubated for 1 h with avidin-biotin complex (ABC Elite kit, Vector laboratories). After rinses the sections were developed in diaminobenzidine for ~2 min (Sigma, D4168), rinsed, and immediately dehydrated and coverslipped with Permount. Images were captured on a Nikon E800 microscope and analyzed using Image J software (NIH) to determine immunoreactive regions. For the GFAP densitometry analysis and microglia counts 2 sections were used per mouse and averaged to generate a single value. To establish the relative microglial size, 6 representative microglia were selected per animal for pixel count and also averaged to generate a single value.

### Quantitative RT-PCR

RT-PCR was performed using RNA extracted from hypothalamus of the experimental mice. After brains were removed, the left brain hemisphere was placed in RNAlater. The hypothalamus was subsequently removed and total RNA was extracted using a homogenizer (Ultra-Turrax T8, IKAWorks, Wilmington, NC) and an RNeasy Mini Kit (Qiagen, Austin, TX) according to manufacturer instructions. RNA was then reverse transcribed into cDNA with M-MLV Reverse Transcriptase enzyme (Invitrogen, Carlsbad, CA). Gene expression for TNFα, IL6, SOCS3, Ikbkb, Nfkbia, MAC1 and POMC were determined using inventoried primer and probe assays (Applied Biosystems, Foster City, CA) on an ABI 7500 Fast Real Time PCR System using Taqman® Universal PCR Master Mix. The universal two-step RT-PCR cycling conditions used were: 50°C for 2 min, 95°C for 10 min, followed by 40 cycles of 95°C for 15 s and 60°C for 1 min. Relative gene expression of individual samples run in duplicate was calculated by comparison to a relative standard curve and standardized by comparison to 18S rRNA signal.

### Liquid chromatography mass spectrometry of oxidized phospholipids

Lipids from brain of mice exposed to FA or PM_2.5_ were extracted three times with chloroform/methanol mixture (1:1) and combined extracts were evaporated to dryness under stream of nitrogen. Samples were stored under nitrogen atmosphere at −80°C until analysis. Mass spectra were acquired in positive ion mode using Applied Biosystems 3200 QTRAP system coupled with electrospray ionization (TurbolonSpray) source. The spectrometer was optimized by infusion of PAPC (25 nmol/ml) and POVPC (5 nmol/ml). All phospholipids were purchased from Avanti Polar Lipids Inc, (Alabaster, AL). The source parameters were set as follows: curtain gas (nitrogen), 10 psi; collision gas (nitrogen), medium; ion spray voltage 5000 V; temperature 550°C, ion spray voltage, 5000 V; ion source gas 1 and 2, 30 and 50 psi, respectively. Optimized parameters for all phospholipids were: declustering potential, 50 V; entrance potential, 10 V and collision energy, 50 eV. For analysis of brain extracts, samples were dissolved in mobile phase consisting of chloroform, methanol, water and trifluoroacetic acid (65:25:4:0.1, by vol). Lipids were characterized after isocratic separation on 5 m Zorbax RX-SIL 4.6 mm × 250 mm HPLC column (Agilent Technologies, Santa Clara, CA) at 0.4 ml/min flow rate using Shimadzu LC-20 AD pump interfaced to a Shimadzu CBM-20A system controller. Mass spectrometer was operated in multiple reactions monitoring (MRM) positive ionization mode. Specific monitor Q1/Q3 ion pairs were m/z 782184 for PAPC and m/z 594184 for POVPC. Standard curves for all phospholipids were obtained in the same set of experiments by infusion of serially diluted PAPC and POVPC. All data were acquired and processed by Analyst software (version 1.4.2, Applied Biosystems, Foster City, CA).

### Data analysis

Data are expressed as means ± standard error of the mean unless otherwise indicated. For the analysis of hypothalamic inflammation biomarkers, linear regression was used with PM exposure, treatment group, and PM x treatment interaction as the independent variables. Based on our previous studies, we had an a priori hypothesis regarding the direction of the outcome. A one-tailed t-test was used to analyze the effect of 5-week or 8-week PM exposure on mRNA expression in hypothalamus. To examine the effect of PM exposure on glucose homeostasis, body weight, and food intake in each treatment group (PM and FA), a stratified analysis using linear regression (with PM exposure, treatment group, and their interaction) was initially performed at each time point of measurement. In addition, an overall repeated measures analysis was performed using all observations with covariates in the linear model: PM, treatment group, PM x treatment interaction, and time. For body weight and food intake, the analysis further adjusted for baseline measures (0 week) when available. For the measurements on energy metabolism/expenditure response, two analysis strategies were used - (1) taking the average over time (cycles) for each mouse and then using linear regression and (2) using repeated measures analysis, adjusting for time. For all repeated measures analyses, in each model a correlation structure was chosen based on the Akaike Information Criterion (AIC) value to account for the correlation between repeated measurements on the same mouse or mice staying in the same cage. A p-value of less than 0.05 was deemed statistically significant. All analyses were performed using R2.15.0.

## Abbreviations

aCSF: artificial cerebrospinal fluid
CNS: Central nervous system
FA: Filtered air
IR: Insulin resistance
ITT: Insulin tolerance test
ICV: Intracerebroventricular
IPGTT: Intra-peritoneal glucose tolerance testing
OxPAPC: Oxidized 1-palmitoyl-2-arachidonoyl-sn-glycero-3-phosphocholine
PM: Particulate matter
PM_2.5_: Particulate matter <2.5 μm in aerodynamic diameter
POMC: Proopiomelanocortin
RER: Respiratory exchanging ratio
SOCS: Suppressor-of-cytokine signaling
HOMA-IR: Homeostasis model assessment of the IR index
Type II DM: Type II diabetes
VAT: Visceral adipose tissue
